# Spectroscopic evaluation of U^VI^–cement mineral interactions: ettringite and hydro­talcite

**DOI:** 10.1107/S1600577521011553

**Published:** 2022-01-01

**Authors:** Antonia S. Yorkshire, Martin C. Stennett, Brant Walkley, Sarah E. O’Sullivan, Lucy M. Mottram, Daniel J. Bailey, John L. Provis, Neil C. Hyatt, Claire L. Corkhill

**Affiliations:** a Immobilization Science Laboratory, Department of Materials Science and Engineering, University of Sheffield, Sheffield S1 3JD, United Kingdom; b Sustainable Materials at Sheffield, Department of Chemical and Biological Engineering, University of Sheffield, Sheffield, United Kingdom

**Keywords:** U^VI^, uranium, actinides, ettringite, hydro­talcite

## Abstract

Portland cement based grouts used for radioactive waste immobilization contain high replacement levels of supplementary cementitious materials, including blast-furnace slag and fly ash. The minerals formed upon hydration of these cements may have capacity for binding actinide elements present in radioactive waste. In this work, the minerals ettringite (Ca_6_Al_2_(SO_4_)_3_(OH)_12_·26H_2_O) and hydro­talcite (Mg_6_Al_2_(OH)_16_CO_3_·4H_2_O) were selected to investigate the importance of minor cement hydrate phases in sequestering and immobilizing U^VI^ from radioactive waste streams. U *L*_III_-edge X-ray absorption spectroscopy (XAS) was used to probe the U^VI^ coordination environment in contact with these minerals. For the first time, solid-state ^27^Al magic angle spinning nuclear magnetic resonance (MAS-NMR) spectroscopy was applied to probe the Al coordination environment in these U^VI^-contacted minerals and make inferences on the U^VI^ coordination, in conjunction with the X-ray spectroscopy analyses. The U *L*_III_-edge XAS analysis of the U^VI^-contacted ettringite phases found them to be similar (>∼70%) to the uranyl oxyhydroxides present in a mixed becquerelite/metaschoepite mineral. Fitting of the EXAFS region, in combination with ^27^Al NMR analysis, indicated that a disordered Ca- or Al-bearing U^VI^ secondary phase also formed. For the U^VI^-contacted hydro­talcite phases, the XAS and ^27^Al NMR data were interpreted as being similar to uranyl carbonate, that was likely Mg-containing.

## Abstract 2

A U *L*_III_-edge X-ray absorption spectroscopic investigation of U^VI^ interactions with minor cement minerals, ettringite and hydro­talcite, is reported. The study is supplemented by ^27^Al magic angle scattering nuclear magnetic resonance spectroscopy, X-ray diffraction and geochemical modelling.

## 1. Introduction

Cementitious binders are used extensively in radioactive waste management. In particular, intermediate- and low-level radioactive waste (ILW and LLW, respectively) are generally suitable for cementitious encapsulation as they are non-heat-generating; in the UK these are encapsulated using cement blends with high replacement levels of Portland cement (PC) by supplementary cementitious materials (SCMs), including blast-furnace slag (BFS) and fly ash (FA) (Ojovan & Lee, 2005 ▸; Batchelor, 2006 ▸). However, ILW streams in particular may still contain measurable radioactivity, some of which will arise from the presence of actinides (Nuclear Decommissioning Authority, 2019 ▸). It is, therefore, imperative that steps are taken to understand the fundamental interactions of actinides with cement materials.

Until recently in the UK, once UO_2_ fuel had been used within a nuclear reactor, it was reprocessed to separate the usable U and Pu (to recycle into new fuel) from highly active fission products. The fuel cladding, when separated from the UO_2_ fuel, is encapsulated in a BFS:PC cement grout (Radioactive Waste Management, 2016 ▸). The recovered U is treated further for fuel fabrication; however, a large surplus of depleted U remains. One of the options for management of this material, comprising ^238^UO_3_ and ^238^U_3_O_8_ powders, is encapsulation within a cement, or mixing with concrete to form a depleted uranium aggregate (DUAGG) which could potentially be used to line vaults in a geological disposal facility (Nuclear Decommissioning Authority, 2014 ▸; Radioactive Waste Management, 2016 ▸). In these scenarios, cement grout will therefore come into direct contact with U-bearing material. Pu-contaminated materials (PCM) arising from fuel reprocessing operations are immobilized in a FA:PC grout (Nuclear Decommissioning Authority, 2013 ▸). Since the primary decay product of Pu is U, cements that encapsulate PCM will, in the future, also contain U isotopes.

In hardened PC blended with SCMs, the microstructure is dominated by a Ca- and Si-rich binder phase known as a calcium–silicate–hydrate phase (‘C–S–H’). Studies to understand the interaction of U with cement materials have therefore predominantly been focused on U^VI^_(aq)_ interactions with C–S–H phases, demonstrating good U^VI^ uptake and/or secondary U^VI^ phase precipitation (Wieland *et al.*, 2010 ▸; Harfouche *et al.*, 2006 ▸). However, within cement matrices, other minor cement hydrate phases can form in conjunction with the C–S–H binder phase, and studies considering the importance of these phases for actinide immobilization are less extensive. Given that alkaline pH ranges will prevail under cementitious conditions, aqueous U speciation will be dominated by uranyl hydroxides [*i.e.* UO_2_(OH)_*x*_^*y*−^] (Sutton *et al.*, 2003 ▸) and interlayer anion-exchange mechanisms may be conceivable for U^VI^ uptake.

**Figure 1:**
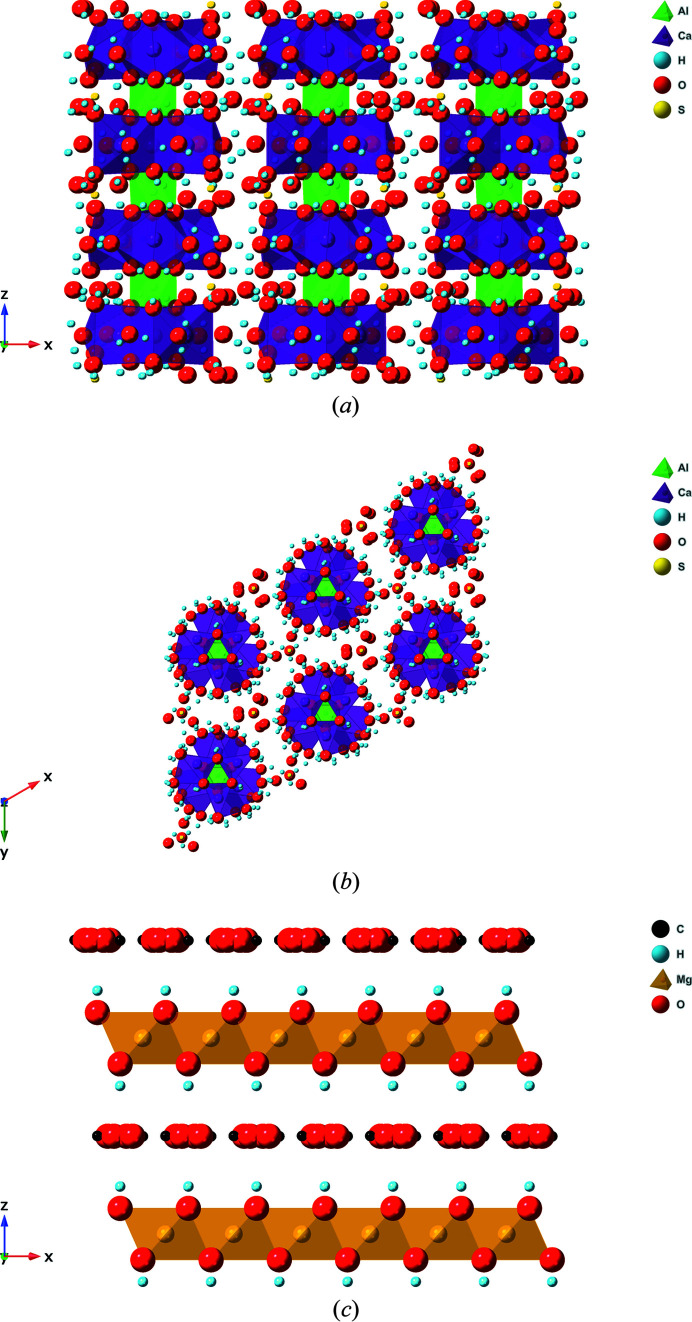
Representation of the ettringite crystal structure shown down the *y*(*b*) axis [panel (*a*)] and the *z*(*c*) axs [panel (*b*)] (Goetz-Neunhoeffer & Neubauer, 2006 ▸). Panel (*c*) shows a ‘cross-sectional’ view of the hydro­talcite LDH structure shown down the *y*(*b*) axis (Radha *et al.*, 2007 ▸), where Mg = Mg or Al. Not to scale.

Ettringite (Ca_6_Al_2_(SO_4_)_3_(OH)_12_·26H_2_O; AFt-SO_4_) is the tri-sulfate phase that forms in PC as a result of hydration of the tri-calcium aluminate (3CaO·Al_2_O_3_) clinker phase in the presence of gypsum (CaSO_4_·2H_2_O) (Bullard *et al.*, 2011 ▸); gypsum is added to cement clinker during production (Hewlett & Liska, 2019 ▸). Ettringite can also form in blends containing BFS or FA, to a certain extent (Lothenbach *et al.*, 2011 ▸). The channel-like structure of ettringite, formed by columns of Al hydroxide and Ca hydroxide polyhedra that incorporate sulfate (SO_4_^2−^) anions [see Figs. 1 ▸(*a*) and 1 ▸(*b*)] (Goetz-Neunhoeffer & Neubauer, 2006 ▸; Clark *et al.*, 2008 ▸), shows potential for anion-exchange and incorporation within its structure. This has been demonstrated previously for anionic radionuclide species, such as pertechnetate (Tc^VII^O_4_^−^), which exchanges in for sulfate in the ettringite channels (Saslow *et al.*, 2020 ▸). Hydro­talcite-type phases (*e.g.* Mg_6_Al_2_(OH)_16_CO_3_·4H_2_O) have been observed to form extensively in cement blends with high BFS contents, and to some extent in those containing FA, as a result of their high Al and moderate Mg content (Lothenbach *et al.*, 2011 ▸; Richardson & Groves, 1992 ▸). Hydro­talcite phases have a layered double hydroxide (LDH) structure [see Fig. 1 ▸(*c*)], an assemblage that may show good ion-exchange properties (Wijitwongwan *et al.*, 2019 ▸), as previously demonstrated for interlayer anions such as chloride (Cl^−^) and carbonate (CO_3_^2−^) (Ke *et al.*, 2017 ▸). However, studies using non-cementitious Zn,Al-based carbonate LDHs have demonstrated a decrease in U^VI^ uptake at pH values above ∼7, coinciding with a release of carbonate interlayer anions into solution and resulting in U^VI^-carbonate aqueous complexation (Pshinko *et al.*, 2013 ▸). Therefore, carbonate-type hydro­talcite LDH phases that form in cement matrices (and thus at higher pH values) may show potential for capture of U^VI^ complexes by surface sorption or secondary phase formation, rather than structural incorporation.

In this study, ettringite and hydro­talcite phases were synthesized and contacted with aqueous U^VI^. The local chemistry and coordination of the secondary U^VI^ phases formed in, or in conjunction with, ettringite and hydro­talcite minerals were probed using U *L*_III_-edge X-ray absorption spectroscopy (XAS). Characterization of the structural modification induced in ettringite and hydro­talcite minerals as a consequence of U^VI^ incorporation was also performed using solid-state ^27^Al magic angle spinning nuclear magnetic resonance (MAS-NMR) spectroscopy.

## 2. Experimental methods

### 2.1. Materials

ACS-grade NaOH (≥97.0%), Na_2_CO_3_ (99%), Al(NO_3_)_3_·9H_2_O (≥98%), Mg(NO_3_)_2_·6H_2_O (99%), Ca(OH)_2_ (≥97.0%) and Al_2_(SO_4_)_3_·16H_2_O (≥98%), supplied by Sigma Aldrich, were used for the synthesis of cement minerals. Ultra high quality deionized water (referred to as UHQ hereafter) was used for all aqueous solutions and suspensions, generated by filtration to achieve a resistivity measurement of 18.18 MΩ cm^−1^. All weighing of precursors was carried out under ambient conditions on the benchtop, but mixing, filtration and storage were carried out under an N_2_ atmosphere to prevent carbonation of cement minerals, unless otherwise stated.

### 2.2. Ettringite and hydro­talcite synthesis

A novel synthesis method was devised for producing ettringite, using hydro­thermal treatment, developed from methods previously reported in the literature (Goetz-Neunhoeffer *et al.*, 2006 ▸; Yang & Guo, 2014 ▸). The stoichiometry of the reaction was based on equation (1):

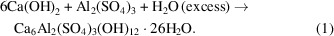




Ca(OH)_2_ was added to an aqueous solution of Al_2_(SO_4_)_3_ in Ar-degassed UHQ in stoichiometric amounts and the solution-suspension was mixed well before it was poured into Teflon-lined Parr vessels which were sealed, tightened and placed into a heating block for 1 week at 180°C (not under N_2_ atmosphere). After reaction, the resulting solids were removed from the Parr vessels and dried at 35°C for ∼24 h before being ground to a fine powder for characterization; they were subsequently stored under N_2_.

A pH-controlled solution mixing method was used to synthesize hydro­talcite, similar to the method reported elsewhere (Aimoz *et al.*, 2012 ▸). A solution of 1 *M* Mg(NO_3_)_3_/1 *M* Al(NO_3_)_3_ was added dropwise to a 1 *M* Na_2_CO_3_ solution, and the pH was maintained at >pH 11.0 with additions of 0.5 *M* NaOH where necessary. This method precipitated an Mg- and Al-containing LDH with a ‘carbonate interlayer’. The precipitated solid was filtered gravitationally using a Whatman-542-ashless filter paper and washed with a minimum of 10 ml UHQ to ensure removal of residual salts and carbonates. The powder was dried under ambient atmosphere at 35°C for ∼24 h before being ground into a fine powder for characterization; it was subsequently stored under N_2_.

### 2.3. U^VI^ contact experiments

Aqueous U^VI^ contact experiments were performed on both ettringite and hydro­talcite cement mineral phases. The dry powders were added to aqueous solutions of U^VI^ in UHQ [as uranyl nitrate; UO_2_NO_3__(aq)_] at concentrations of both 0.5 m*M* (‘borderline trace’) and 10 m*M* (‘elevated’), achieving a solids-to-liquid ratio of 25 g l^−1^. The suspensions were mixed on a rotary shaker for 48 h, after which time they were filtered through 0.22 µm cellulose filters. The solution pH values were measured before the solutions were acidified and prepared for ICP-OES (inductively coupled plasma optical emission spectrometry) analysis (ThermoFisher iCAP Duo 6300) to measure U, Ca, S, Al or Mg concentrations. The remaining solids were dried at ambient temperature, under N_2_, for at least 24 h before preparation for X-ray diffraction (XRD), XAS and MAS-NMR spectroscopy. Table 1 ▸ displays the sample designations, and the target U^VI^ loading per mineral phase.

#### 2.3.1. Geochemical modelling estimations

Geochemical modelling was performed using the *Phreeqc Interactive 3.4.0-12927* software and the Lawrence Livermore National Laboratory thermodynamic database, to estimate the saturation index (SI) of mineral phases likely to form in aqueous solution under the experimental conditions of the U^VI^ contact studies. The results from ICP-OES analyses and the solution pH values were used for the model input for Ca, S, Al or Mg, while the U^VI^ concentration corresponded to either the 0.5 m*M* or 10 m*M* concentration in the initial solution.

### 2.4. Solid-state analysis

XRD measurements of all ettringite and hydro­talcite phases were performed both before and after U^VI^ contact experiments, using a Bruker D2 desktop instrument. Powders were compressed into a 10 mm-diameter recess on a low-background Si(111) plate in a PMMA holder. For U-containing samples (*i.e.* after U^VI^ contact) the compressed powder was covered with an acetate film held in place with a small amount of PVA adhesive, in accordance with alpha-powder handling protocols. Measurements were taken between 5 and 50° 2θ for ettringite samples and 5 and 70° 2θ for hydro­talcite samples. The counting time was 1 s per step, in increments of 0.02° 2θ with a 1 mm divergence slit.

U *L*_III_-edge (17166 eV) XAS was performed at Diamond Light Source (DLS) (on beamline B18) to obtain information in the XANES and EXAFS regions of each of the U^VI^-contacted ettringite and hydro­talcite samples, as well as for a suite of standard U-bearing mineral and ceramic phases (see Table 2 ▸), in transmission mode. The amount of material required to allow for transmission measurement at one absorption length was calculated using the *Hephaestus* program (Ravel & Newville, 2005 ▸); for U^VI^-contacted mineral phases this was estimated based on the known general chemical formula of the mineral phases and an assumption of 100% U^VI^_(aq)_ uptake from solution. The accurately weighed powders were pressed into pellets using a polyethyl­ene glycol (PEG) binder (∼50 mg) to allow for mechanical stability, pressed at ∼1 tonne for ∼1 min.

A Si(111) monochromator with beam collimation (achieved using a Cr- and Pt-coated Si mirror) was utilized (Diaz-Moreno *et al.*, 2018 ▸). An Y foil was used in the reference channel for monochromator calibration. The *Athena* program was used for post-processing and normalization of data (Ravel & Newville, 2005 ▸). Data calibration was performed by assigning the first inflection point of the derivative energy spectrum (*i.e.* *E*_0_) for the Y foil in the reference channel as 17038 keV (*K* edge) (Bearden & Burr, 1967 ▸). The value of *E*_0_ for each data set was then assigned to the position of the maximum inflection point of its derivative energy spectrum.

Linear combination fitting analysis was applied to the XANES region of the spectra using the *Athena* software. A combination of any two of the considered phases (Table 2 ▸) was allowed to be fitted within the region of −20 and +30 eV from the position of *E*_0_. The value of Δ*E* for each phase fit was recorded. The ‘best fit’ for each sample was chosen based on a combination of prior knowledge of the system deduced from XRD, NMR, geochemical modelling estimations, in addition to *R*-factor and χ^2^ values.

The *Artemis* program was used for the generation of scattering pathways and fitting of models for the EXAFS region (Ravel & Newville, 2005 ▸). In *Athena*, prior to this, the fitting window for the Fourier transform of *k* space into *R* space was selected where the signal in *k* space was approximately equal to 0, using a Hanning window (d*k* = 0), before being imported into *Artemis*. Scattering paths were generated using *FEFF* (Ravel & Newville, 2005 ▸) calculations of appropriately selected CIF files as the input, using prior knowledge of the system determined from XRD and geochemical modelling estimations as a starting point. Pathways were fitted between ∼1 and ∼5 Å in *R* space using a Hanning window (d*R* = 0). Δ*E* was allowed to vary as a global parameter. As well as single scattering (SS) pathways, multiple scattering (MS) pathways were considered for U–O_ax_–O_ax_ (linear) or U–O_ax_–O_eq_ (linear) interactions, where applicable (see Section 3.3).

The value of the amplitude reduction factor (*S*_0_^2^) for a U absorber measured on beamline B18 (DLS) was determined in the model for UO_2_ as 0.86, using pathways generated from the CIF file for UO_2_ (ICSD No. 160814) (Greaux *et al.*, 2008 ▸), and was thereafter fixed in the model for the fitting of all other phases. The first-shell coordination number for U^VI^-contacted mineral phases was determined by setting *S*_0_^2^ in the model and allowing the product of (*N*_*X*1_ × *S*_0_^2^) to vary, where *N*_*X*1_ is the first-shell coordination number.

The U^VI^-contacted minerals were also measured by solid-state ^27^Al MAS-NMR spectroscopy, as well as pure-phase ettringite and hydro­talcite for comparison. Samples were packed into 4 mm ZrO_2_ sample rotors and spectra were collected using a Bruker Avance III HD 500 spectrometer at 11.4 T, with a resulting Larmor frequency of 130.32 MHz for ^27^Al. ^27^Al chemical shifts were referenced to Al(NO_3_)_3 (aq)_. A magic angle spinning (MAS) rate of 12.5 kHz was applied. Conventional single-pulse experiments were carried out using an optimized pulse length of 1.4 µs and recycle delays of 35 s and 25 s for ettringite and hydro­talcite systems, respectively. A total of 256 scans were acquired for each sample. Post-processing of the data was carried out using the *TopSpin 4.0.6* software, and data were normalized by integrated area.

## 3. Results

### 3.1. U^VI^ uptake by ettringite and hydro­talcite

**Figure 2:**
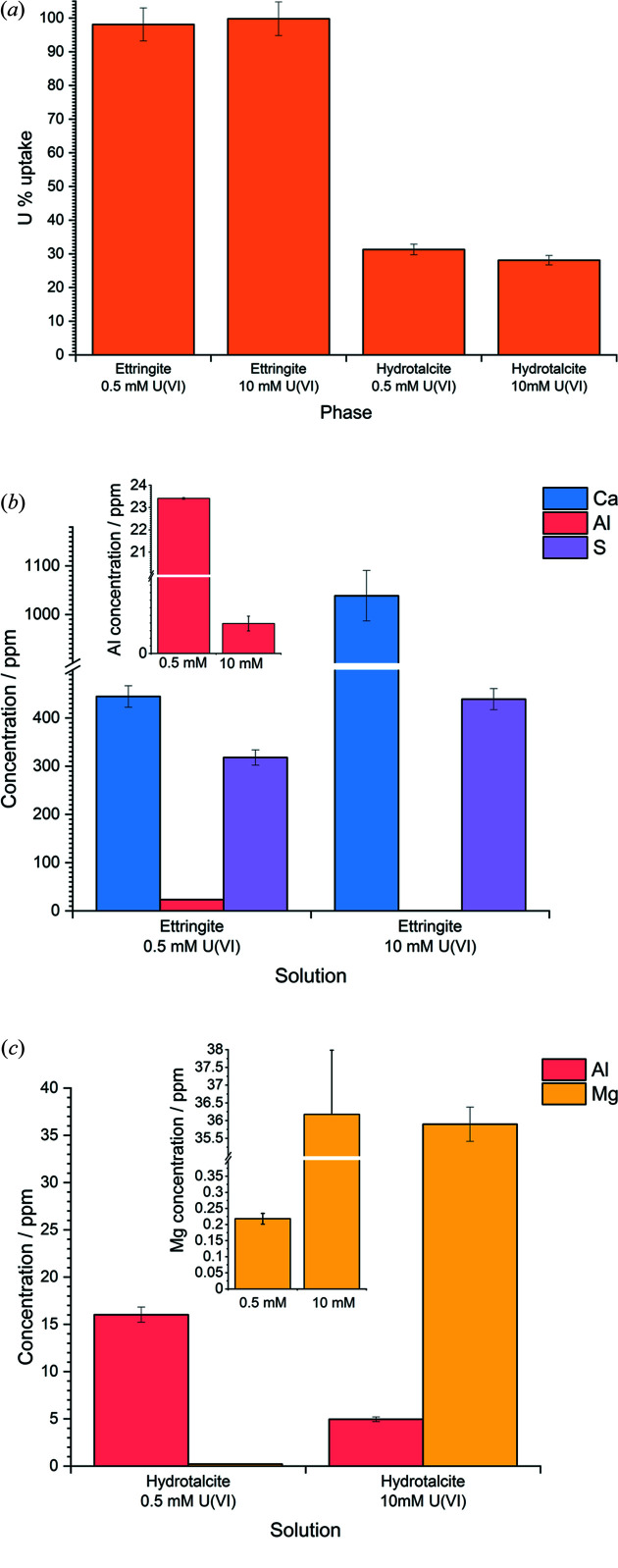
Aqueous elemental analysis of solutions containing U^VI^ and cement minerals. (*a*) U^VI^ removal from solution by ettringite and hydro­talcite as a percentage of [U^VI^]_*t* = 0_; (*b*) Ca, Al and S release from ettringite in contact with U^VI^; (*c*) Al and Mg release from hydro­talcite in contact with U^VI^. Error bars represent one standard deviation of triplicate samples.

The pH measurements for the 0.5 m*M* and 10 m*M* U^VI^ solutions both before and after contact with ettringite and hydro­talcite are given in Table 3 ▸. The removal of U^VI^ from solution (*i.e.* U^VI^ uptake by the solid) as a percentage of [U^VI^]_*t*=0_ by both ettringite and hydro­talcite is shown in Fig. 2 ▸(*a*). At both 0.5 m*M* and 10 m*M* U^VI^, ettringite effectively showed complete uptake of U^VI^ (>99%) whereas hydro­talcite showed ∼30% uptake at both U^VI^ concentrations. It was concluded that the discrepancy in the uptake between the two mineral phases was due to the significant amount of dissolved carbonate released from the hydro­talcite phases, leading to U^VI^-carbonate complex formation in solution, increasing the U^VI^ solubility and thus decreasing the amount of U^VI^ uptake by the solid phase (Pshinko *et al.*, 2013 ▸).

The release of Ca, Al, S or Mg from ettringite and hydro­talcite into solution is given in Figs. 2 ▸(*b*) and 2 ▸(*c*), respectively. Ca and S release from ettringite was shown to increase with increasing U^VI^ concentration, thus with a decrease in pH. The release of Ca was over two times higher after contact with 10 m*M* U^VI^ compared with 0.5 m*M* U^VI^ ([Ca] = ∼1000 ppm versus ∼450 ppm), whereas the S concentration showed a smaller increase ([S] = ∼300 ppm versus ∼400 ppm). For hydro­talcite, the Mg release was very low in the 0.5 m*M* [U^VI^] solution, at 0.20 ± 0.02 ppm, and was significantly increased in the 10 m*M* U^VI^ solution, at 36.2 ± 1.8 ppm. This observed leaching was insufficient to significantly alter the minerals, which retained their crystallographic structure (see Section 3.2).

The Al release in both ettringite and hydro­talcite displayed the opposite behaviour, *i.e.* the concentrations decreased with increasing U^VI^ concentration (*i.e.* with decreasing pH). For hydro­talcite, this could be related to the decrease in Al solubility with decreasing pH at the two different U^VI^ concentrations (pH ∼10.2 compared with pH ∼7.8). For ettringite, this could also be the case to a certain extent, with a pH decrease from ∼10.5 to ∼9.5; however, the explanation for this could be more complex and requires the justifications of XRD and NMR analyses (see Sections 3.3 and 3.4).

### 3.2. Phase analysis before and after U^VI^ contact

**Figure 3:**
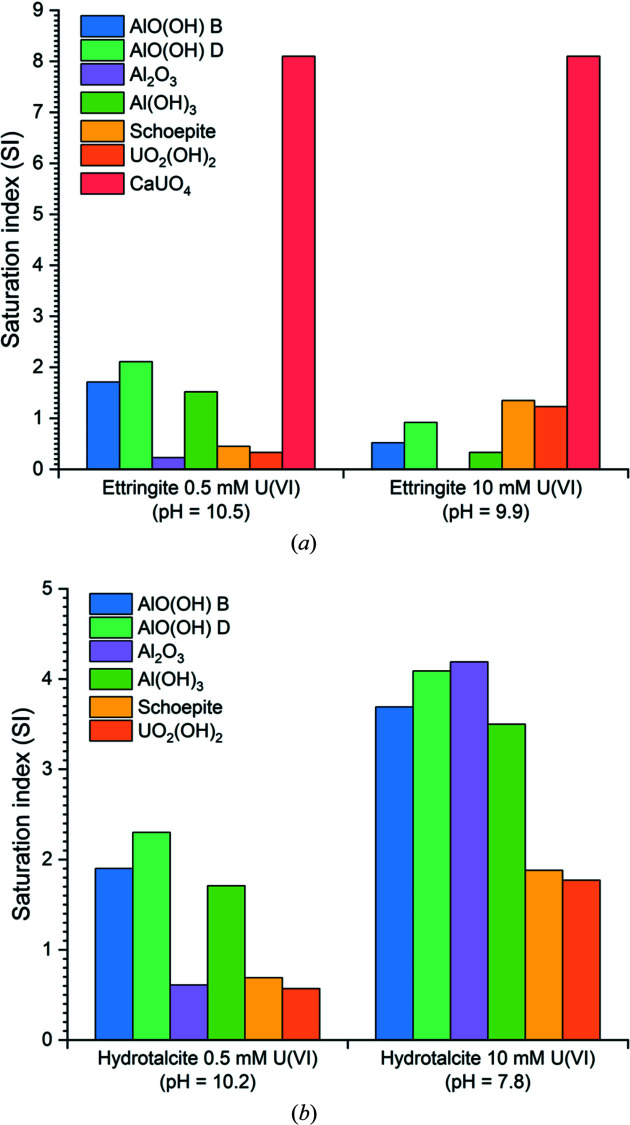
Geochemical modelling results showing all estimated saturated phases over the experimental pH range measured for the (*a*) ettringite and (*b*) hydro­talcite samples, in contact with 0.5 m*M* U^VI^ and 10 m*M* U^VI^.

The concentrations of Ca, S, Al or Mg released into solution and measured pH (Table 3 ▸) were input modelled using *Phreeqc*, at U^VI^ concentrations of 0.5 m*M* (119 ppm) and 10 m*M* (2380 ppm), to ascertain the thermodynamically feasible Ca-, S-, Al-, Mg- and/or U-containing saturated phases in the corresponding systems (Fig. 3 ▸).

Boehmite and diaspore (AlO(OH) polymorphs, denoted as ‘B’ and ‘D’, respectively), corundum (Al_2_O_3_), gibbsite (Al(OH)_3_), metaschoepite (UO_3_·2H_2_O) and uranium hydroxide (UO_2_(OH)_2_) were identified as being saturated in both hydro­talcite-U^VI^ systems [Fig. 3 ▸(*b*)]. For the ettringite-U^VI^ systems, calcium uranate (CaUO_4_) was additionally identified; however, at the 10 m*M* U^VI^ concentration, Al_2_O_3_ had a negative saturation index, likely due to the low concentration of Al measured in solution [Fig. 3 ▸(*a*)]. Although the calcium uranate phase identified in this system is a high-temperature phase (Takahashi *et al.*, 1993 ▸), hydrous forms of calcium uranate exist (*e.g.* CaU_2_O_7_·*x*H_2_O_(cr)_) and calcium uranate phases are typically solubility limiting for U/Ca at high pH (Finch & Ewing, 1997 ▸; Valsami-Jones & Ragnarsdöttir, 1997 ▸; Sutton *et al.*, 2003 ▸; Ding *et al.*, 2016 ▸; Ding, 2017 ▸; Çevirim-Papaioannou *et al.*, 2018 ▸; Yalçıntaş *et al.*, 2019 ▸; Adam *et al.*, 2021 ▸).

**Figure 4:**
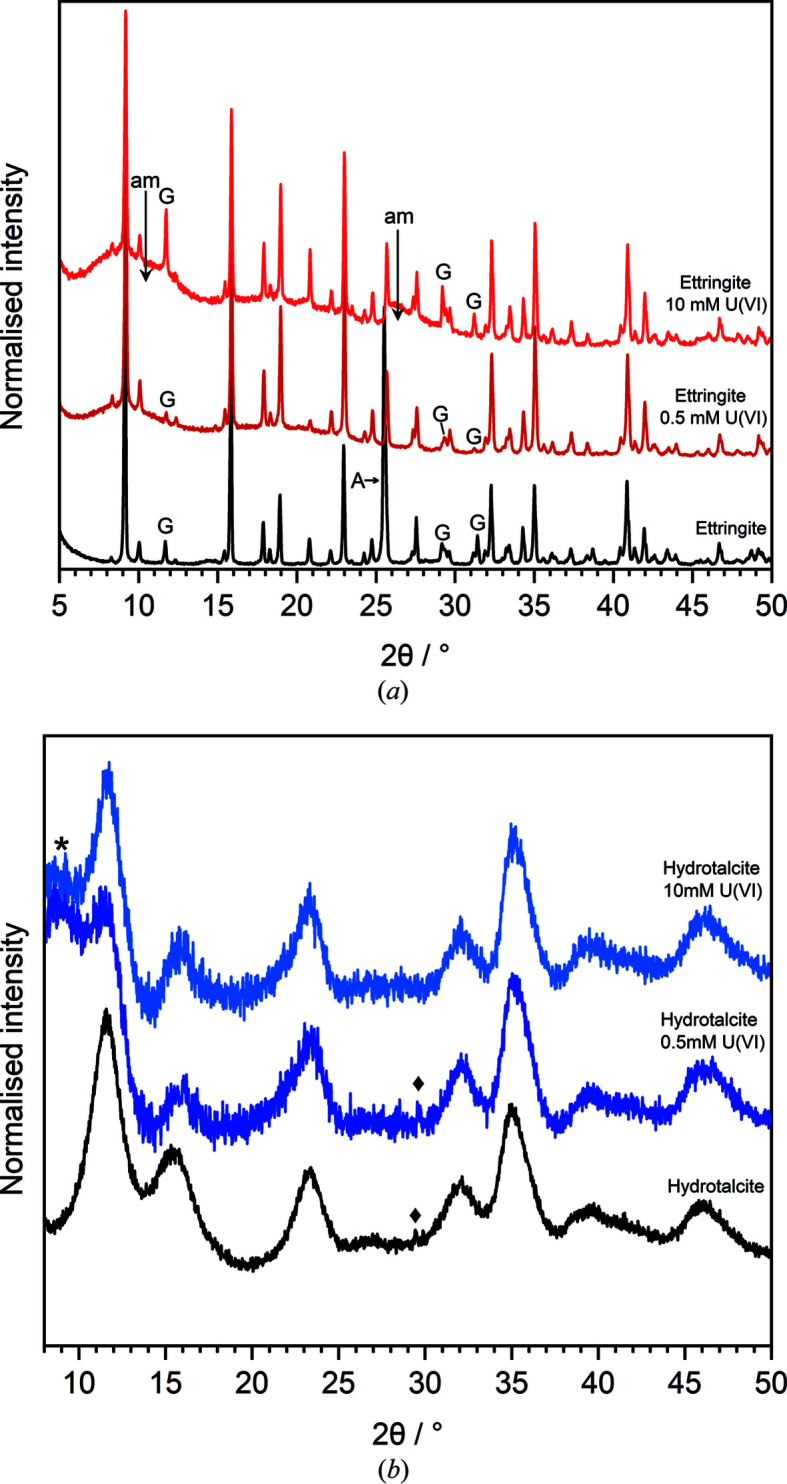
XRD patterns of (*a*) ettringite [PDF No. 04-013-3691 (Goetz-Neunhoeffer *et al.*, 2006 ▸)] and (*b*) hydro­talcite [PDF No. 01-082-8041 (Taylor, 1973 ▸)], before and after contact with U^VI^ at 0.5 m*M* and 10 m*M*. A = anhydrite [CaSO_4_; PDF No. 00-037-1496 (McMurdie *et al.*, 1986 ▸)]; G = gypsum [CaSO_4_·2H_2_O; PDF No. 00-033-0311 (Morris *et al.*, 1980 ▸)]. Diffraction patterns are normalized to the maximum peak intensity in both systems. In (*a*), regions of diffuse scattering are a result of sample preparation methods for radioactive samples unless denoted by ‘am’. In (*b*), background subtraction was performed on the diffraction patterns to highlight the diffuse diffraction peaks. The black diamond symbol indicates an unidentified phase and the asterisk indicates the background from sample preparation methods that could not be subtracted.

The geochemical modelling predictions were evaluated upon XRD analysis of the U^VI^-contacted phases (Fig. 4 ▸). The reflections assigned to the originally synthesized ettringite phase [powder diffraction file (PDF) No. 04-013-3691] (Goetz-Neunhoeffer *et al.*, 2006 ▸) were still present in the samples contacted with 0.5 m*M* and 10 m*M* U^VI^ solution [Fig. 4 ▸(*a*)]. The peak attributed to the reflection of anhydrite [CaSO_4_; PDF No. 00-037-1496 (McMurdie *et al.*, 1986 ▸)] at ∼25.5° 2θ (denoted ‘A’) disappeared at both U^VI^ concentrations, likely due to the dissolution of anhydrite by the low-pH uranyl nitrate solution. The peaks assigned to gypsum [CaSO_4_·2H_2_O; PDF No. 00-033-0311 (Morris *et al.*, 1980 ▸); denoted ‘G’] decreased in intensity, relative to ettringite, after contact with 0.5 m*M* U^VI^ but increased in intensity after contact with 10 m*M* U^VI^, which is unexpected given the corresponding increase in Ca and S released to solution. This therefore suggests increased ettringite dissolution in the 10 m*M* U^VI^ solution, compared with gypsum dissolution. The lower concentration of Al in the 10 m*M* U^VI^ solution could, therefore, be explained by the precipitation of a poorly crystalline secondary Al hydroxide phase, potentially indicated by the regions of diffuse scattering observed between ∼7–13° and ∼26–30° 2θ (denoted ‘am’), that could also be U-containing. Given that >99% uptake of U^VI^ was observed and no other U-containing phases were identifiable by XRD, it is certainly plausible that these amorphous regions may arise from a poorly crystalline U-containing phase. ^27^Al MAS-NMR analysis on these solid phases was used to evaluate these hypotheses (see Section 3.4).

The XRD peaks for nanocrystalline hydro­talcite [PDF No. 01-082-8041 (Taylor, 1973 ▸)] were maintained on addition of both 0.5 m*M* and 10 m*M* U^VI^ solutions [Fig. 4 ▸(*b*)]. The diffuse nature of these diffraction patterns makes the identification of any low-yield secondary phases challenging; however, there appears to be little to no change in the diffraction pattern on addition of both concentrations of U^VI^. In the starting phase a peak at ∼29.5° 2θ was partially indexed as boehmite [AlO(OH); PDF No. 01-074-2895 (Bokhimi *et al.*, 2001 ▸)]. This phase was also present in the 0.5 m*M* U^VI^-contacted sample but not in the 10 m*M* U^VI^-contacted sample. In both the ettringite and hydro­talcite systems, there was no obvious (*i.e.* XRD observable) identification of the mineral phases indicated by the corresponding geochemical modelling.

### 3.3. Local coordination chemistry of U^VI^ associated with ettringite and hydro­talcite

**Figure 5:**
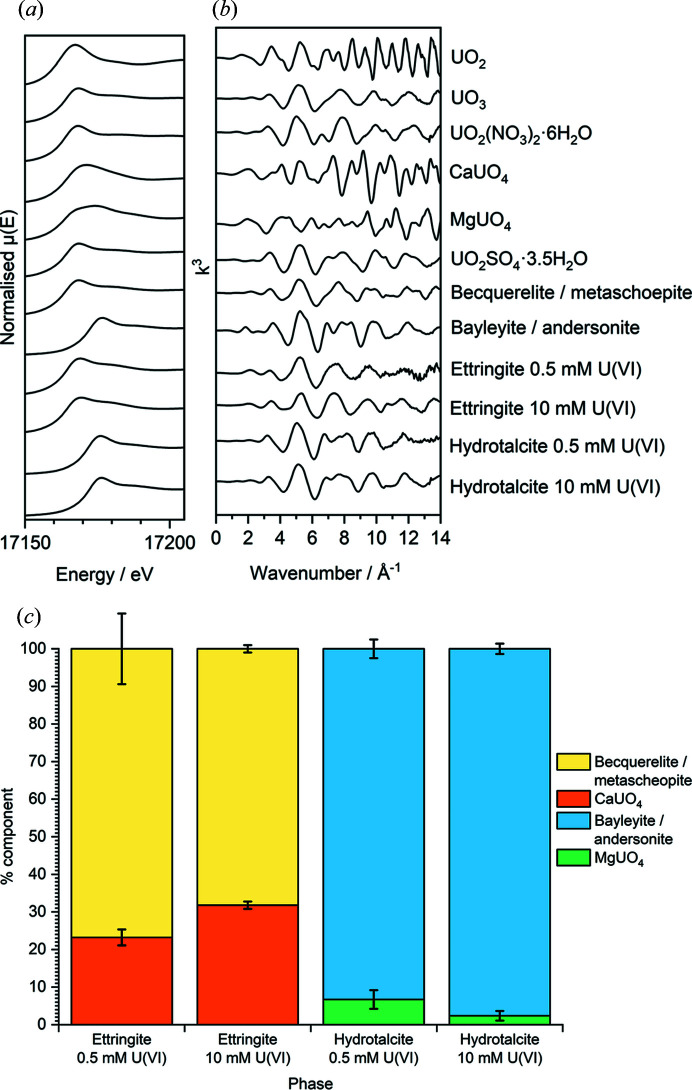
U *L*_III_-edge energy spectra of all U^VI^-contacted phases and mineral standards, showing (*a*) the XANES region (normalized and offset for clarity); (*b*) the corresponding *k* ^3^-weighted EXAFS spectra (offset for clarity); and (*c*) a bar graph highlighting the percentage component of signals contributing to the linear combination fit of each system (graphical fits are given in the supporting information).

The U *L*_III_-edge energy XANES spectra and *k* ^3^-weighted spectra for the standard U-bearing mineral and ceramic phases and the U^VI^-contacted ettringite and hydro­talcite minerals are shown in Figs. 5 ▸(*a*) and 5 ▸(*b*), respectively, along with the percentage composition of XANES signals contributing to the linear combination fits for the U^VI^-contacted ettringite and hydro­talcite systems in Fig. 5 ▸(*c*). The results for the weighted component for each linear combination fit are also given in Table 4 ▸.

The linear combination fit for the XANES region of the U^VI^-contacted ettringite phases did not alter significantly as a function of U^VI^ concentration. The XANES signals were comparable largely with those of the mixed becquerelite/metaschoepite mineral phase, at ∼77% and ∼70% for 0.5 m*M* and 10 m*M* U^VI^ solutions, respectively. The remainder of the signals showed a contribution similar to that of calcium uranate (CaUO_4_) in both cases. Signal domination from the becquerelite/metaschoepite phase indicates the retention of the uranyl moiety, that may be bonded to Ca, in addition to the co-formation of a calcium uranate type phase.

The linear combination fit for the XANES region of the U^VI^-contacted hydro­talcite phases also showed a similar pattern irrespective of U^VI^ concentration. The majority of the XANES signals were comparable with those of the mixed bayleyite/andersonite phase, at >90% for both concentrations of U^VI^. The small remainder of the signals were comparable with those of magnesium uranate (MgUO_4_) in both cases. This is indicative of the formation of a uranyl carbonate phase, which may be bonded to Mg, in addition to the co-formation of a magnesium uranate type phase.

**Figure 6:**
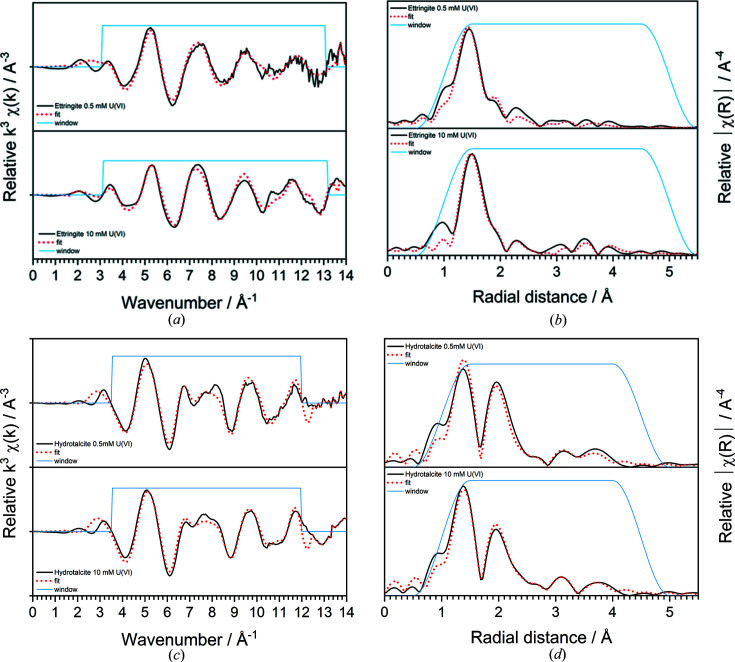
Local coordination analysis of U^VI^ in contact with cement minerals. (*a*) *k* ^3^-weighted spectra and model fit (dashed red lines) for ettringite; (*b*) corresponding Fourier-transformed radial plots; (*c*) *k* ^3^-weighted spectra and model fits (dashed red lines) for hydro­talcite; (*d*) corresponding Fourier-transformed radial plots and fits.

The *k* ^3^-weighted spectra and radial distribution profiles of the U^VI^-contacted ettringite and hydro­talcite minerals, and subsequent EXAFS model fits for each, are shown in Fig. 6 ▸, with the fit parameters given in Table 5 ▸. Additionally, fit parameters for the mixed bayleyite/andersonite and mixed becquerelite/metaschoepite mineral phases are given.

For U^VI^-contacted ettringite systems, the Fourier transform window was set between *k* = ∼3 and *k* = ∼13 Å^−1^. A combination of *FEFF* pathways was generated using the CIF files for metaschoepite [((UO_2_)_4_O(OH)_6_)(H_2_O)_5_; ICSD No. 156714] and becquerelite [Ca((UO_2_)_6_O_4_(OH)_6_)(H_2_O)_8_; ICSD No. 94620] (Burns & Li, 2002 ▸; Klingensmith *et al.*, 2007 ▸). For ettringite contacted with 0.5 m*M* U^VI^, the O_ax_ distance was refined at 1.827 ± 0.012 Å with *N*_O1_ = 2.9 ± 0.3. A split equatorial shell was evident by fitting subsequent O_eq_ pathways refined at distances of 2.23 ± 0.02, 2.34 ± 0.02, 2.45 ± 0.03 and 2.90 ± 0.04 Å with *N*_O2_ = 1, *N*_O3_ = 2, *N*_O4_ = 1 and *N*_O5_ = 1, respectively. A Ca scatterer was also fitted at 3.62 ± 0.07 Å with *N*_Ca1_ = 1, by refining the pathway generated for the U–Ca distance in becquerelite. A subsequent U distance was also fitted at 3.80 ± 0.06 Å with *N*_U1_ = 1. It should be noted that the data for this phase were not well resolved after ∼10 A^−1^ in *k*, as demonstrated in Fig. 6 ▸(*a*).

When the model used for the 0.5 m*M* U^VI^-ettringite data was applied to the 10 m*M* U^VI^-ettringite EXAFS data, it yielded a poor fit. Rather, a different model was devised that contained no U–Ca pathway. Instead, a U–C pathway was fitted, generated using the CIF file for andersonite [Na_2_Ca(UO_2_(CO_3_)_3_)·*x*(H_2_O); ICSD No. 15533] (Coda *et al.*, 1981 ▸). This is suggestive of carbonation of the phase, likely from some unavoidable CO_2_ ingress during storage or measurement, leading to coordination of U to C. The first O_ax_ distance was refined at 1.837 ± 0.008 Å with *N*_O1_ = 1.7 ± 0.2. A split equatorial shell was also evident by fitting subsequent O_eq_ pathways refined at distances of 2.24 ± 0.01, 2.38 ± 0.02, 2.53 ± 0.03 Å with *N*_O2_ = 2, *N*_O3_ = 2, *N*_O4_ = 1, respectively; however, these fitted distances were closer than with the 0.5 m*M* system. The C distance was refined at 2.91 ± 0.05 Å with *N*_C1_ = 1, with two subsequent U scatterers also fitted at distances of 3.74 ± 0.03 and 3.90 ± 0.03 Å, both with *N*_U1,2_ = 1. It should be noted that the MS pathways considered for U–O_ax_–O_ax_ or U–O_ax_–O_eq_ were not included in the fit for either of the U^VI^-contacted ettringite minerals, due to the expected low-symmetry geometry of U^VI^ in the phase formed, as discussed further in Section 4.1.

For U^VI^-contacted hydro­talcite systems, the Fourier transform window was set between *k* = ∼3.5 and *k* = ∼12 Å^−1^. A combination of *FEFF* pathways was generated using the CIF files for bayleyite [Mg_2_(UO_2_(CO_3_)_3_)·18H_2_O; ICSD No. 32101] and magnesium ortho­uranate [Mg(UO_2_)_2_; ICSD No. 24725] (Zachariasen, 1954 ▸; Mayer & Mereiter, 1986 ▸). For hydro­talcite contacted with 0.5 m*M* U^VI^, the O_ax_ distance was refined at 1.814 ± 0.009 Å with *N*_O1_ = 2.5 ± 0.2. The subsequent O_eq_ pathway was refined at 2.44 ± 0.01 Å with *N*_O2_ = 4. A C scatterer was fitted at a distance of 2.93 ± 0.02 Å with *N*_C1_ = 3, by refining the pathway generated for the U–C distance in bayleyite. A U scatterer was also fitted at 3.39 ± 0.03 Å with *N*_U1_ = 2, by refining the pathway generated for the U–U distance in magnesium orthouranate. A subsequent Mg distance was also fitted at 3.83 ± 0.02 Å with *N*_Mg1_ = 4.

It was possible to fit the 10 m*M* U^VI^-contacted hydro­talcite data with the same model, and the distances refined were the same within error. The value of *N*_O1_ refined for O_ax_ was slightly increased at 2.8 ± 0.2. It should be noted that an Al scatterer at the same distance in place of Mg also yielded a similar fit and *R* factor; however the results from ^27^Al MAS-NMR analyses justify the fitting of Mg in this case (see Section 3.4).

The U–O_ax_–O_ax_ MS pathway was also fitted at approximately twice the distance of *R* for the SS U–O_ax_ pathway in both the U^VI^-contacted hydro­talcite phases. The contribution to the fit was minor in both cases, and contributions at *R* > 3 Å were largely dominated by Mg and U single scatterers rather than the MS pathway.

### 3.4. Influence of U^VI^ on the chemical environment of Al

**Figure 7:**
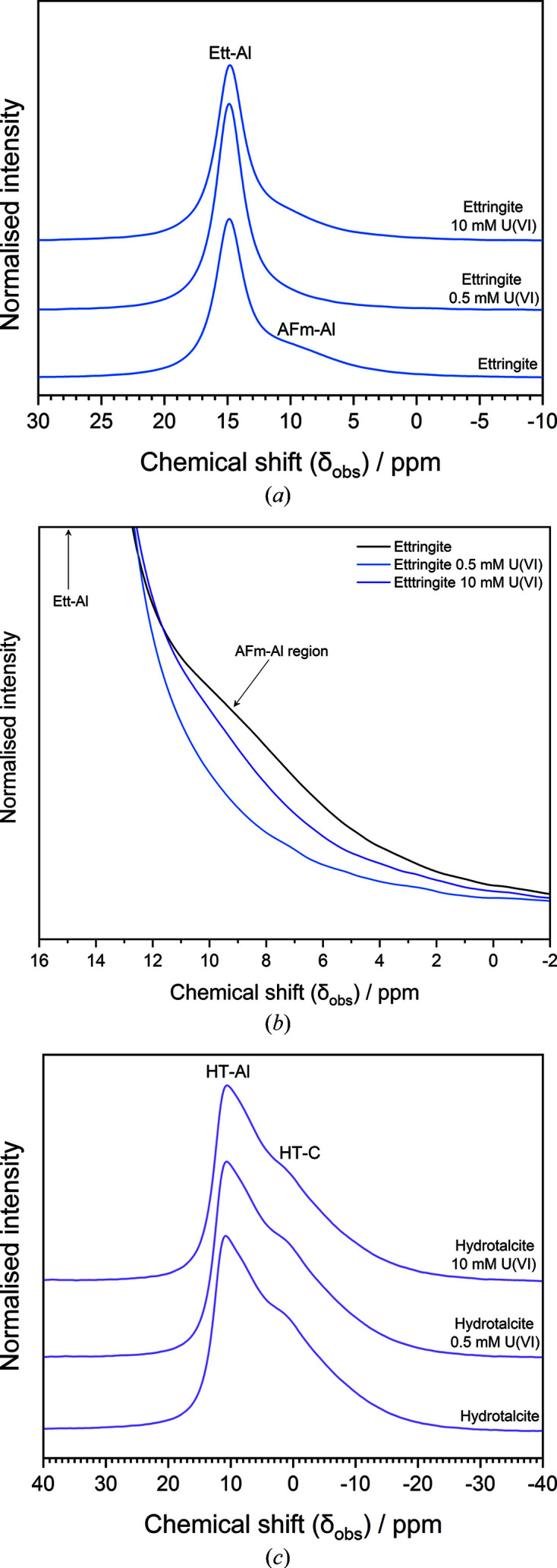
^27^Al MAS-NMR (*B*_0_ = 11.7 T, ν_*R*_ = 12.5 kHz) spectra of (*a*) pristine ettringite and ettringite contacted with 0.5 m*M* and 10 m*M* U^VI^; (*b*) the same data as (*a*) but highlighting the ‘AFm-Al’ region of the spectra; and (*c*) pristine hydro­talcite and hydro­talcite contacted with 0.5 m*M* and 10 m*M* U^VI^.

The normalized ^27^Al MAS-NMR spectra of pure-phase and U^VI^-contacted ettringite phases are shown in Fig. 7 ▸(*a*). The main peak exhibited an observed chemical shift (δ_obs_) at δ_obs_ = 15 ppm, which arises from the two octahedrally coordinated Al sites in ettringite that cannot be further resolved at the magnetic field used in this study (9.4 T) (Skibsted *et al.*, 2017 ▸). These sites are denoted as ‘Ett-Al’. There is an additional small, broad peak at δ_obs_ = ∼10 ppm present in the pure-phase ettringite phase, appearing as a shoulder of the main ettringite peak, which is attributed to octahedrally coordinated Al in calcium aluminate mono­sulfate phases [AFm, Ca_4_(Al_2_O_6_)(SO_4_).12H_2_O]. This arises from minor impurities of this phase, remnant from the synthesis process and not detectable by XRD. This site is denoted as ‘AFm-Al’ (Skibsted *et al.*, 1993 ▸).

The peak arising from octahedral Al in ettringite was maintained after contact of the phase with U^VI^ at both concentrations [Fig. 7 ▸(*a*)]. This is consistent with the retention of diffraction peaks for ettringite in the corresponding XRD patterns. However, the shoulder for the octahedral Al sites in AFm was only observed in the pristine sample and the phase contacted with 10 m*M* U^VI^, albeit at a slightly lower intensity than in the pristine mineral phase [Fig. 7 ▸(*b*)]. This behaviour could be attributed to two possible scenarios, given that Al release into solution was higher for the 0.5 m*M* U^VI^ sample when compared with the 10 m*M* U^VI^ sample: (i) increased dissolution of the impurity AFm phase at 0.5 m*M* U^VI^ and/or higher retention at 10 m*M* U^VI^; or (ii) the precipitation of a poorly crystalline or low-yield U-substituted AFm phase in the 10 m*M* U^VI^-contacted sample, given that AFm is a LDH that can display ion-exchange capabilities (Aimoz *et al.*, 2012 ▸).

The normalized ^27^Al MAS-NMR spectra of pristine and U^VI^-contacted hydro­talcite phases are shown in Fig. 7 ▸(*c*). The spectra displayed a peak at δ_obs_ = 11 ppm, which is attributed to the single octahedrally coordinated Al environment in hydro­talcite that is surrounded by octahedrally coordinated Mg atoms (Walkley & Provis, 2019 ▸; Sideris *et al.*, 2012 ▸). This peak is denoted as ‘HT-Al’. There was also a shoulder observed at δ = 1–3 ppm in all spectra, which arises due to shielding of some of the Al atoms due to the presence of CO_3_^2−^ interlayer anions in hydro­talcite (denoted as ‘HT-C’) (Walkley & Provis, 2019 ▸; Sideris *et al.*, 2012 ▸). The observation of these peaks at all concentrations of U^VI^ contact is consistent with the retention of diffuse diffraction peaks for hydro­talcite in the corresponding XRD patterns. There was no notable change observed in the spectra as a result of U^VI^ contact at both concentrations, which suggests that no significant solid-phase structural interaction of U^VI^ with Al within the hydro­talcite phase occurred.

## 4. Discussion

### 4.1. U^VI^-ettringite systems

In the ettringite system, the XRD peaks for ettringite were maintained upon contact with both concentrations of U^VI^. However, identification of an amorphous region in the XRD pattern could be the result of a poorly crystalline Al- or Ca- and U^VI^-bearing phase. Geochemical modelling estimations and previous literature allude to the formation of a calcium uranate type phase as the most highly saturated U^VI^-bearing phase, due to the abundance of Ca in the system.

The results from the XANES region linear combination fitting analyses indicate that the XANES region was largely dominated by a signal similar to that of the mixed becquerelite/metaschoepite mineral phase at both concentrations of U^VI^. This indicates that the uranyl moiety [O=U=O]^2+^ was maintained, and with reference to the EXAFS model fits this is likely to be in a pentagonal bipyramidal coordination given that a total of *N*_Oeq_ = 5 were fitted for samples of ettringite exposed to 0.5 m*M* and 10 m*M* U^VI^. This is also consistent with the U^VI^ geometry found in becquerelite (Colmenero *et al.*, 2018 ▸) (see also the EXAFS model fit for mixed becquerelite/metaschoepite in Table 5 ▸). In uranyl compounds that display this low-symmetry coordination geometry, it has been shown that contributions from MS pathways are very minor and do not contribute significantly to spectral features (Thompson *et al.*, 1997 ▸). This was evident when performing the fits and accounts for the exclusion of the MS pathways for the U^VI^-contacted ettringite phases (and becquerelite/metaschoepite mineral). Whilst signal contribution from a calcium uranate type environment was also indicated by XANES linear combination fitting, it is likely that this would form in this system as a hydrous analogue [*e.g.* CaU_2_O_7_·*x*H_2_O_(cr)_] (Çevirim-Papaioannou *et al.*, 2018 ▸). Calcium uranate phases have previously been found to form in cementitious systems (Sutton *et al.*, 2003 ▸; Felipe-Sotelo *et al.*, 2017 ▸).

Although the EXAFS model fits of the two U^VI^-contacted ettringite systems varied most notably by inclusion/exclusion of Ca/C scattering atoms, this is thought to be a result of unavoidable carbonation of the 10 m*M* U^VI^-contacted ettringite phase during preparation or analysis. In this case, the fitting of a C scatterer suggests the formation of a uranyl carbonate type phase, whereby U^VI^ could be coordinated to a carbonate ligand. Such a phase would thus display a XANES signal that is not easily distinguishable from that of a becquerelite-type phase or the 0.5 m*M* phase. However, the EXAFS model interpretation must be treated with some caution, as C and Ca are relatively low-*Z* atoms and may not display a large contribution to the EXAFS signal, especially in such disordered and multi-phase systems. The fitting of U scatterers in both cases was a strong indication that a secondary U^VI^ precipitate was formed.

From the ^27^Al NMR data, a significant change in the main peak corresponding to the octahedrally coordinated Al sites in ettringite was not observed as a result of U^VI^ contact. This suggests that there was no incorporation of U^VI^, for example, into the columnar channels of the ettringite structure, even at the ‘borderline trace’ (*i.e.* sorption-controlled) concentration of U^VI^ (0.5 m*M*). It is thought that a close proximity of U^VI^ to Al hydroxide polyhedra in this way would result in a downward shift in δ_obs_, as a result of increased shielding of Al nuclei by U. Considering these observations, it seems plausible that at both concentrations of U^VI^ a poorly crystalline surface or secondary precipitate containing Ca would be partly responsible for the sequestration of U^VI^ in an ettringite-only system.

The presence of an AFm-SO_4_ (Ca_4_(Al_2_O_6_)(SO_4_)·*x*H_2_O) impurity in the ettringite phase was indicated by the shoulder on the main ettringite peak, at ∼10 ppm by ^27^Al MAS-NMR. This peak was shown to diminish for the sample contacted with 0.5 m*M* U^VI^, but it was evident for the sample contacted with 10 m*M* U^VI^. This behaviour coincides with the relative leaching of Al from ettringite upon contact with the low-pH uranyl nitrate bearing solution [Fig. 2 ▸(*b*)], *i.e.* the 0.5 m*M* U^VI^-contacted sample released more Al into solution than the 10 m*M* U^VI^-contacted sample; therefore, in the former, the AFm-SO_4_ phase was not retained, while in the latter it was. However, since the relative leaching of Ca and S was higher in the 10 m*M* U^VI^ solution than in the 0.5 m*M* U^VI^ solution, due to the lower pH of the former (pH ∼2.8 and ∼3.6, for 10 m*M* U^VI^ and 0.5 m*M* U^VI^, respectively), if one assumes that in the 10 m*M* U^VI^ solution ettringite was leached more than in the 0.5 m*M* U^VI^ solution, then the discrepancy in Al leaching may be attributed to the formation of a secondary Al phase that incorporates U^VI^, potentially AFm-U^VI^.

While it has been shown that AFt-SO_4_ (*i.e.* ettringite) phases have capacity for the uptake of anionic species such as pertechnetate (TcO_4_^−^) (Saslow *et al.*, 2020 ▸), evidence for the same behaviour in AFm-SO_4_ phases is more limited. One example is that of iodate (I^−^), which has been shown to incorporate into the interlayer of AFm-SO_4_ to form an AFm phase with a mixed sulfate and iodate interlayer (Aimoz *et al.*, 2012 ▸). The results from geochemical modelling performed in the present study indicated the presence of uranyl hydroxide/sulfate anions in solution at the pH values of the ettringite solutions (pH ∼10), including: (UO_2_)_3_(OH)_7_^−^, UO_2_(OH)_3_^−^, UO_2_(OH)_4_^2−^ and UO_2_(SO_4_)_2_^2−^. The precipitation of an AFm-U^VI^ phase is therefore plausible, with the higher concentration of U^VI^ in the 10 m*M* solution, and thus higher U^VI^ uptake is required for formation and/or detection of this phase by NMR.

From the species identified by geochemical modelling, the UO_2_(OH)_4_^2−^ and UO_2_(SO_4_)_2_^2−^ anions are the most likely candidates that could directly exchange into an AFm-SO_4_ interlayer, potentially forming ‘Ca_4_(Al_2_O_6_)(UO_2_(OH)_4_).*x*H_2_O’- and ‘Ca_4_(Al_2_O_6_)(UO_2_(SO_4_)_2_).*x*H_2_O’-type phases, respectively, taking into account the charge balance. However, given that the ionic radius of a sulfate anion (SO_4_^2−^) is 2.42 Å (Marcus, 1988 ▸), the aforementioned uranyl hydroxide or uranyl sulfate anion combinations would encompass a much larger ionic radius [*e.g.* OH^−^ = 1.1 Å (Marcus, 2012 ▸); UO_2_^2+^ ≃ 0.95 Å (Dean *et al.*, 2008 ▸)]. An anion-exchange process may therefore be size limited and identification of such a phase is speculative without further evidence from analysis of a solely U^VI^-exchanged AFm-SO_4_ phase.

### 4.2. U^VI^-hydro­talcite systems

For the U^VI^-contacted hydro­talcite systems, no significant change in the XRD pattern was observed when compared with the pristine hydro­talcite. However, approximately 30% of [U^VI^]_*t* = 0_ was removed from solution at both concentrations of U^VI^, indicating that U^VI^ was sequestered by the solid phase to some extent.

The results from the XANES linear combination fitting and EXAFS model fitting of the U^VI^-contacted hydro­talcite systems indicated that the coordination of U^VI^ was largely unchanged as a function of U^VI^ concentration. A large contribution to the XANES signal linear combination fit in both cases was attributed to the mixed bayleyite/andersonite mineral phase. This indicates that the uranyl moiety [O=U=O]^2+^ was maintained. With reference to the EXAFS model fit, the value of *N*_O2_ = 4 for O_eq_ agrees with the value of *N*_O2_ for that of the uranyl carbonate mixed bayleyite/andersonite mineral phase. These minerals both display a hexagonal bipyramidal U^VI^ geometry (Mayer & Mereiter, 1986 ▸; Coda *et al.*, 1981 ▸). A small signal contribution from magnesium uranate in both cases was also indicated in the XANES region; as with calcium uranate, it is likely that this phase would be hydrous in nature [*e.g.* MgU_2_O_7_·*x*H_2_O_(cr)_] (Yalçıntaş *et al.*, 2019 ▸).

The O_ax_, O_eq_ and C distances obtained in the EXAFS model fits for the U^VI^-contacted hydro­talcite systems are also fairly typical of a uranyl carbonate phase. The distances are summarized in Table 6 ▸, and compared with the values obtained for the mixed bayleyite/andersonite phase (EXAFS model fits given in Table 5 ▸) and for those reported by van Veelen *et al.* (2018 ▸) for brucite (Mg(OH)_2_), hydro­magnesite (Mg_5_(CO_3_)_4_(OH)_2_·4H_2_O) and nesquehonite (MgCO_3_·3H_2_O) minerals contacted with a 2 m*M* uranyl nitrate solution at ∼34 g l^−1^.

In the results obtained by van Veelen *et al.* (2018 ▸), Mg and U scatterers were fitted at distances of ∼3.6 Å and ∼3.9 Å, respectively. This is in contrast to the result obtained here for hydro­talcite, where Mg and U were fitted conversely at ∼3.8 and ∼3.4 Å, respectively. This could be a result of the U^VI^ coordination having mixed magnesium uranate character in conjunction with the formation of a uranyl carbonate phase, whereby the scattering U atom is at a closer scattering distance in magnesium uranate phases, compared with its relative position in a uranyl carbonate phase only (Zachariasen, 1954 ▸).

The ^27^Al MAS-NMR spectra for hydro­talcite are unchanged as a result of U^VI^ addition to hydro­talcite which indicates that Al did not play a role in the uptake and coordination of U^VI^, therefore supporting the concept of Mg as the scattering atom in the EXAFS model. This is consistent with the formation of a U^VI^,Mg,C-containing surface precipitate or sorbed species, rather than anion-exchange into the hydro­talcite interlayer. This observation is also in agreement with the work of van Veelen *et al.* (2018 ▸), who proposed that U^VI^ was sorbed to the surface of Mg-bearing minerals as an outer-sphere complex. The sequestration of U^VI^ by carbonate in this case is highly conceivable given the presumed abundance of carbonate released into solution by the hydro­talcite phases, and the fact that carbonate has a high affinity for U^VI^ complexation (Sutton *et al.*, 2003 ▸).

### 4.3. Implications for waste disposal

For the ettringite systems, uptake of U^VI^ directly by the ettringite phase was not apparent. Rather, the formation of a poorly crystalline hydrous Ca-containing phase was more plausible. In addition to this, uptake by an AFm impurity contained within the system seemed a more likely mechanism for U^VI^ structural uptake. Like ettringite, AFm-SO_4_ phases are prominent LDH phases that are present in cement matrices, particularly for blast-furnace slag containing blends. Therefore, these should be further investigated to understand their capacity for uptake of U^VI^. Further to this, the development of calcium sulfoaluminate (CSA) cements shows potential for applications in radioactive waste management, and such cements will encompass high levels of ettringite and AFm-SO_4_ phases (Zhou *et al.*, 2006 ▸). Understanding the role that sulfate-containing minerals play in sequestration of highly mobile actinides is therefore pertinent to underpinning the effectiveness of cement blends used for radioactive waste encapsulation, both now and in the future.

The hydro­talcite phases also displayed uptake of U^VI^ through formation of what was concluded to be a surface-sorbed uranyl magnesium carbonate phase, similar to the mineral phase bayleyite, but potentially with some mixed magnesium uranate character. Mg- and Al-containing cement blends, where hydro­talcite forms during hydration, may therefore show good sequestration of aqueous U^VI^ if it is immobilized by association to carbonate and Mg. These findings could also be important if Mg(OH)_2_ sludge wastes are to be immobilized using a cement binder in the future; the high concentration of Mg associated with such sludges derived from the UK Magnox programme will likely lead to formation of significant deposits of hydro­talcite-type LDH minerals within the cement matrix (Walling *et al.*, 2014 ▸), which will be able to effectively immobilize U^VI^ also present in the waste itself.

## 5. Conclusions

Consideration of minor cement hydrate phases for the sequestration of U^VI^ in cement matrices has not been widely reported. Here, for the first time we have probed the solid-state chemistry of ettringite and hydro­talcite minerals that have been subjected to aqueous solutions of U^VI^ using XRD, U *L*_III_-edge XAS and ^27^Al MAS-NMR; these studies are relevant to understanding radioactive waste disposal of actinide-containing materials in cements.

Ettringite phases showed >99% uptake of U^VI^ from solution. Although direct incorporation of U^VI^ into the ettringite structure was not observed in this case, the abundance of Ca in the ettringite systems was likely responsible for sequestering U^VI^ within a Ca-bearing uranyl oxyhydroxide phase or as a hydrous calcium uranate type phase, as indicated by XAS results. This is in agreement with the previous studies that have determined Ca to be solubility limiting for U^VI^ in high-Ca (*i.e.* cementitious) systems (Sutton *et al.*, 2003 ▸; Felipe-Sotelo *et al.*, 2017 ▸). ^27^Al MAS-NMR results indicated that there was no change in the Al coordination environment in the ettringite structure; however, the presence of, or formation of, an AFm-SO_4_ phase that may incorporate U^VI^ was alluded to by changes in the ‘AFm-Al’ region of the NMR spectrum. Further investigation into the sorption and anion-exchange capacity of AFm phases for U^VI^_(aq)_ is required.

Hydro­talcite phases displayed some limited U^VI^ uptake (∼30%) and XAS results indicated that this was attributed to the formation of a precipitated or sorbed uranyl carbonate phase. ^27^Al MAS-NMR results showed that there was no observable change in the Al coordination environments in the hydro­talcite phases, leading to the conclusion that the uranyl carbonate phase was a Mg-containing uranyl carbonate phase. This highlights the importance of carbonate in LDH and/or cementitious systems for sequestering U^VI^, a scenario that is corroborated by the strong tendency of carbonate to complex U^VI^ and form uranyl carbonate species.

## Supplementary Material

Local coordination analysis of U(VI) in becquerelite/metaschoepite and bayleyite/andersonite using EXAFS. DOI: 10.1107/S1600577521011553/yw5003sup1.pdf

## Figures and Tables

**Table 1:** Details of the U^VI^-contacted cement mineral samples

Mineral	U^VI^ aqueous concentration (m*M*)	Target U^VI^ loading on mineral phase (ppm)	Sample designation
Ettringite	0.5	4800	Ettringite 0.5 m*M* U^VI^
Ettringite	10	95000	Ettringite 10 m*M* U^VI^
Hydro­talcite	0.5	4800	Hydro­talcite 0.5 m*M* U^VI^
Hydro­talcite	10	95000	Hydro­talcite 10 m*M* U^VI^

**Table 2:** U-bearing ceramic and mineral phases measured by U *L*_III_-edge XAS

Standard (general formula)	Synthetic (S) or natural (N)	Approximate U oxidation state
Uranium dioxide (UO_2_)†	S	4
Uranium trioxide (UO_3_)	S	6
Uranyl nitrate (UO_2_(NO_3_)_2_·6H_2_O)	S	6
Calcium uranate (CaUO_4_)	S	6
Magnesium uranate (MgUO_4_)	S	6
Uranyl sulfate (UO_2_SO_4_·3.5H_2_O)	S	6
Becquerelite (Ca(UO_2_)_6_O_4_(OH)_6_·8H_2_O)/metaschoepite (UO_3_·*x*H_2_O (*x*<2))‡	N	6
Bayleyite (Mg_2_(UO_2_)(CO_3_)_3_·18H_2_O)/andersonite (Na_2_Ca(UO_2_)(CO_3_)_3_·6H_2_O))‡	N	6

† UO_2_ was used for energy alignment and determination of *S*_0_^2^.

‡ This standard was determined to be a mixture of two phases upon XRD analysis. Both are stated (Yorkshire, 2020 ▸).

**Table 3:** pH measurements of uranyl nitrate starting solution and of solutions where uranyl nitrate was mixed with solid cement mineral phases in solution The errors represent the standard deviation of triplicate measurements.

Solution	pH
0.5 m*M* [U^VI^] solution	3.6 ± 0.1
10 m*M* [U^VI^] solution	2.8 ± 0.0
Ettringite 0.5 m*M* U^VI^	10.5 ± 0.2
Ettringite 10 m*M* U^VI^	9.9 ± 0.2
Hydro­talcite 0.5 m*M* U^VI^	10.2 ± 0.2
Hydro­talcite 10 m*M* U^VI^	7.8 ± 0.2

**Table 4:** Weighted fraction of signal contributions in the XANES region, determined using linear combination fitting, for U^VI^-contacted ettringite and hydro­talcite systems

Mineral phase	U^VI^ concentration (m*M*)	*R* factor	Total weighting	Uranyl phase†		Uranate phase‡	
				Weight	Δ*E*	Weight	Δ*E*
Ettringite	0.5	4 × 10^−4^	1.034	0.794 (97)	0.3 (3)	0.240 (22)	0.07 (33)
	10	3 × 10^−4^	1.013	0.691 (10)	0.03 (4)	0.322 (10)	1.18 (8)
Hydro­talcite	0.5	2 × 10^−3^	1.000	0.933 (25)	0.90 (7)	0.067 (25)	−1.6(1.2)
	10	5 × 10^−4^	1.013	0.989 (14)	−0.12 (4)	0.024 (13)	−0.8(2.0)

† Becquerelite/metaschoepite for ettringite and bayleyite/andersonite for hydro­talcite.

‡ CaUO_4_ for ettringite and MgUO_4_ for hydro­talcite.

**Table 5:** EXAFS model parameters for U^VI^-contacted ettringite and hydro­talcite minerals and mixed becquerelite/metaschoepite and bayleyite/andersonite mineral phases Numbers with no errors have been fixed in the model. *R* = effective interatomic distance, *N* = coordination number, σ^2^ = Debye–Waller factor.

Mineral	[U^VI^] (m*M*)	*R* factor	Δ*E* (eV)	Scatterer	*R* (Å)	*N*	σ^2^
Ettringite	0.5	0.020	10 (2)	O_ax_	1.827 (12)	2.9 (3)	0.005 (1)
				O_eq_	2.23 (2), 2.34 (2), 2.48 (4), 2.90 (4)	1, 2, 1, 1	0.005 (1)
				Ca	3.64 (8)	1	0.015 (11)
				U	3.82 (6)	1	0.010 (7)
	10	0.022	11 (2)	O_ax_	1.837 (8)	1.7 (2)	0.001 (1)
				O_eq_	2.24 (1), 2.38 (2), 2.53 (3)	2, 2, 1	0.001 (1)
				C	2.91 (5)	1	0.004 (6)
				U	3.74 (3)	1	0.002 (3)
				U	3.90 (3)	1	0.002 (3)
Hydro­talcite	0.5	0.018	8 (1)	O_ax_ †	1.814 (09)	2.5 (2)	0.0034 (8)
				O_eq_	2.44 (1)	4	0.0034 (8)
				C	2.93 (2)	3	0.001 (2)
				U	3.39 (3)	2	0.004 (2)
				Mg	3.83 (2)	4	0.001 (2)
	10	0.009	9 (1)	O_ax_ †	1.815 (9)	2.8 (2)	0.0048 (8)
				O_eq_	2.43 (1)	4	0.0048 (8)
				C	2.92 (2)	3	0.002 (2)
				U	3.40 (2)	2	0.004 (2)
				Mg	3.85 (2)	4	0.002 (2)
Becquerelite/metaschoepite‡	–	0.017	9 (2)	O_ax_	1.765 (23), 1.889 (27)	1.4 (4), 1	0.0002
				O_eq_	2.14 (1), 2.30 (1), 2.44 (2), 2.51 (2)	1, 2, 1, 1	0.0002
				U	3.89 (2)	1	0.004 (1)
				U	4.60 (3)	1	0.004 (1)
Bayleyite/andersonite‡	–	0.023	9 (2)	O_ax_ †	1.796 (8)	2.5 (3)	0.0031 (7)
				O_eq_	2.43 (1)	4	0.0031 (7)
				C	2.91 (2)	3	0.01 (2)
				Na	3.71 (5)	1	0.0001
				Ca	3.98 (2)	2	0.001 (2)
				O	4.31 (9)	2	0.0031 (7)

† MS pathways also fitted at approximately twice the O_ax_ distance.

‡ Graphical fits are shown in the supporting information.

**Table 6:** Summary of the U–*X* distances obtained by EXAFS model fitting of 0.5 m*M* and 10 m*M* U^VI^-contacted hydro­talcite, bayleyite/andersonite and U^VI^-contacted magnesium and/or carbonate mineral phases Note that errors were not quoted by van Veelen *et al.* (2018 ▸).

Phase	O_ax_ (Å)	O_eq_ (Å)	C (Å)	Mg (Å)	U (Å)	Reference
Hydro­talcite 0.5 m*M* U^VI^	1.82 (1)	2.44 (1)	2.91 (1)	3.83 (3)	3.39 (3)	This work
Hydro­talcite 10 m*M* U^VI^	1.819 (8)	2.44 (1)	2.93 (1)	3.85 (2)	3.41 (2)	This work
Bayleyite/andersonite	1.796 (9)	2.43 (12)	2.91 (2)	–	–	This work
Brucite + U^VI^	1.80	2.38, 2.48	2.90†	3.60	3.88	van Veelen *et al.* (2018 ▸)
Hydro­magnesite + U^VI^	1.81	2.43	2.90	3.62	3.90	van Veelen *et al.* (2018 ▸)
Nesquehonite + U^VI^	1.81	2.40, 2.50	2.90	3.59	3.89	van Veelen *et al.* (2018 ▸)

† C was thought to be present due to the formation of UO_2_(CO_3_)_3_^4−^ in solution under the ambient experimental conditions adopted by van Veelen *et al.* (2018 ▸).
