# Supplementary material for: Spectroscopic evaluation of UVI–cement mineral interactions: ettringite and hydro­talcite
Source: J Synchrotron Radiat. 2022 Jan 1;29(Pt 1):89–102. doi: 10.1107/S1600577521011553 (PMC8733996; doi:10.1107/S1600577521011553)
Supplement: Supplementary file 1 [file s-29-00089-sup1.pdf]

JOURNAL OF
SYNCHROTRON
RADIATION

Volume 29 (2022)

Supporting information for article:

Spectroscopic evaluation of U^{VI}–cement mineral interactions: ettringite and hydrotalcite

Antonia S. Yorkshire, Martin C. Stennett, Brant Walkley, Sarah E. O'Sullivan, Lucy M. Mottram, Daniel J. Bailey, John L. Provis, Neil C. Hyatt and Claire L. Corkhill

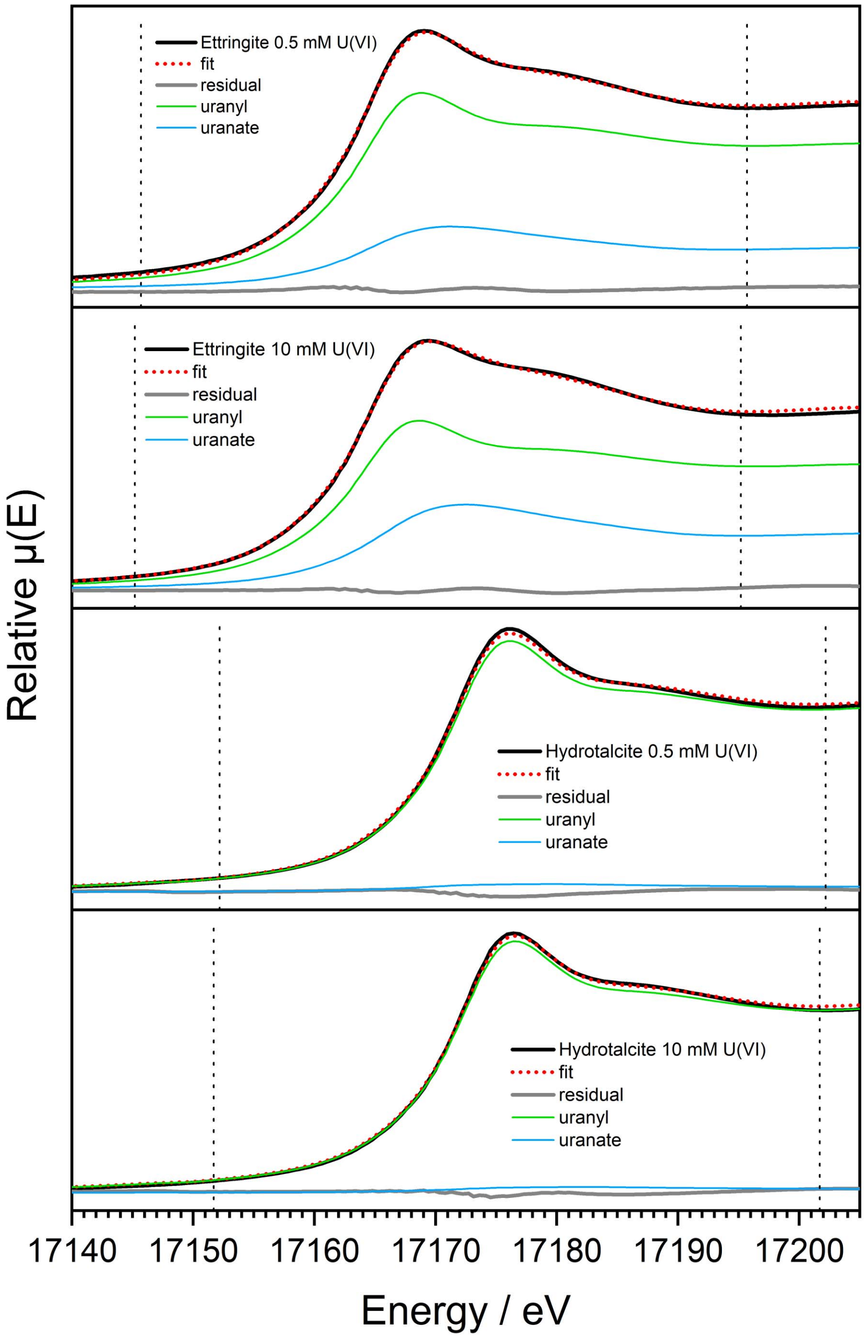

Figure SI1: Graphical fits of linear combination analysis for U(VI)-contacted ettringite and hydrotalcite phases. Dashed black lines indicate the fitted regions.

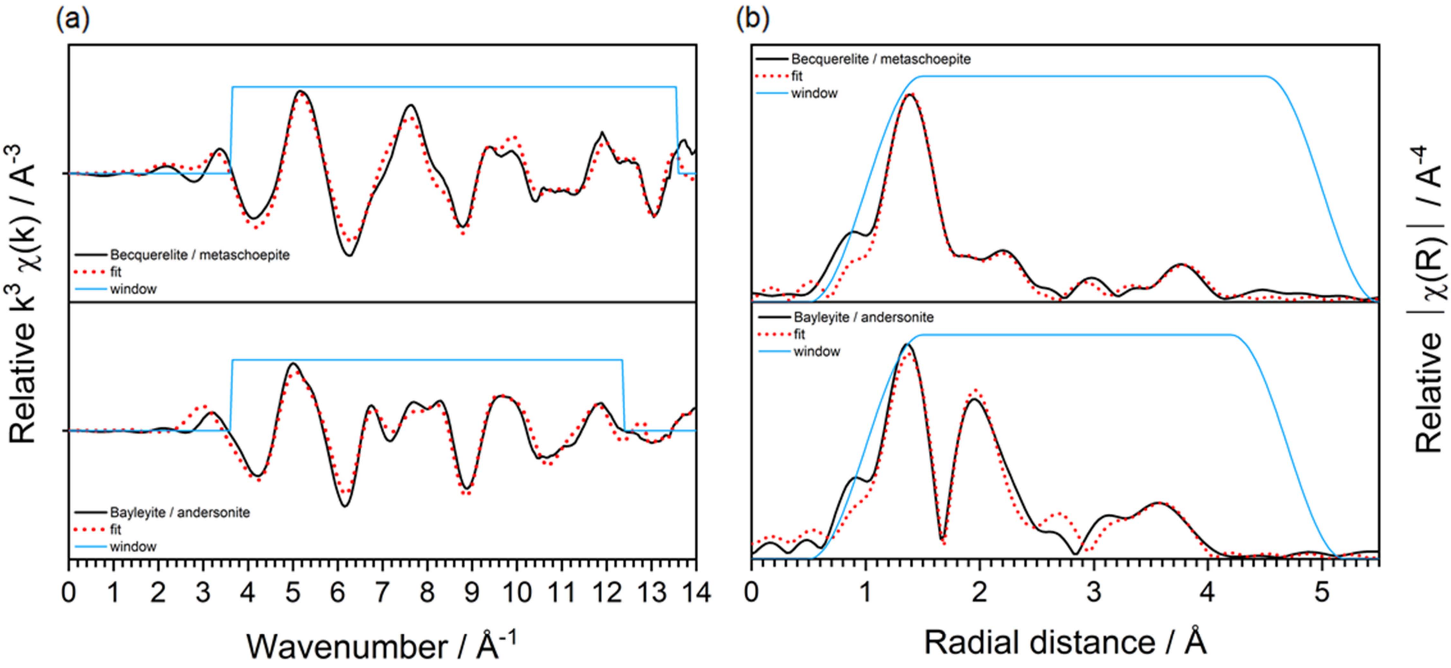

Figure SI2: Local coordination analysis of U(VI) in becquerelite / metaschoepite and bayleyite / andersonite. **(a)** k^3-weighted spectra and model fits (dashed red lines); and **(b)** corresponding Fourier transformed radial plots and fits.
